# Bipolar resection versus enucleation of the prostate in management of benign prostatic hyperplasia patients with large-sized prostates: a prospective randomized controlled clinical trial

**DOI:** 10.1186/s12894-025-02004-1

**Published:** 2026-01-24

**Authors:** Mostafa M. Mostafa, Ahmed F. Ibrahim, Ahmed M. Moeen, Mohammed A. Elgammal, Ahmed S. El-Azab, Mohammad A. Faragallah

**Affiliations:** Assiut University Hospitals and Faculty of Medicine, Assiut, Egypt

**Keywords:** Benign prostatic hyperplasia, Prostate transurethral resection, TURP, Transurethral prostatectomy.

## Abstract

**Background:**

Transurethral resection of the prostate (TURP) remains the standard surgical treatment for benign prostatic hyperplasia (BPH), but its efficacy decreases in large prostates. Bipolar enucleation of the prostate (BipolEP) has been introduced as a modification enabling complete adenoma removal and potentially superior outcomes. This study compared the efficacy and safety of bipolar TURP (B-TURP) and BipolEP in patients with large prostate volumes (≥ 80 mL).

**Patients and methods:**

Seventy patients with prostate volume ≥ 80 mL and bladder outlet obstruction were prospectively randomized to undergo either B-TURP (*n* = 37) or BipolEP (*n* = 33). Both procedures were performed using standardized bipolar platforms. Postoperative management followed uniform bladder irrigation and catheterization protocols. Primary outcomes were functional parameters (IPSS, Qmax, PVR), while secondary outcomes included perioperative parameters and complications. ANCOVA analysis adjusting for baseline IPSS and Qmax, and Post-hoc power analysis were performed.

**Results:**

Both groups showed significant postoperative improvement in IPSS, Qmax, and PVR (*p* < 0.001). Compared with B-TURP, BipolEP achieved a greater reduction in IPSS (*p* = 0.04) and higher postoperative Qmax (*p* = 0.004). Operative time, irrigation volume, catheterization duration, and residual tissue were all significantly lower in the BipolEP group (*p* < 0.05). Retrograde ejaculation occurred more frequently after BipolEP (81.8% vs. 48.6%, *p* = 0.004), whereas other complications were comparable. ANCOVA confirmed that between-group differences in IPSS and Qmax remained significant after adjusting for baseline values.

**Conclusion:**

In patients with large prostates, BipolEP offers superior functional outcomes and improved perioperative efficiency compared with B-TURP, though at a higher risk of retrograde ejaculation. These findings support BipolEP as a preferred surgical option for large-volume BPH.

**Trial registration:**

The study is registered in clinical trials (ClinicalTrials gov ID: NCT05330156 ; Registered on April 15, 2022).

**Supplementary Information:**

The online version contains supplementary material available at 10.1186/s12894-025-02004-1.

## Introduction

Transurethral resection of the prostate (TURP) has traditionally been a standard treatment for benign prostatic hyperplasia (BPH), but its efficacy in large prostates is limited due to longer resection time, bleeding, and incomplete adenoma removal [1]. In contrast, transurethral enucleation of the prostate has emerged as a new standard for managing large glands, as it allows complete adenoma removal while minimizing complications [2]. Both procedures can be performed using bipolar energy, which reduces the risk of transurethral resection syndrome (TURS) and improves intraoperative safety [3]. In this context, we designed a randomized controlled clinical trial to directly compare bipolar TURP (B-TURP) with bipolar enucleation of the prostate (BipolEP) in management of patients with large-volume prostates (≥ 80 mL). The objective was to evaluate perioperative outcomes, functional improvements, and complication rates, providing evidence for optimal surgical selection.

## Patients and methods

### Ethical approval and patient selection

After approval by the Assiut University Faculty of Medicine Institutional Review Board (IRB number: 17200747), we conducted a prospective randomized controlled clinical trial involving 70 BPH patients with large-sized prostates. Inclusion criteria included a diagnosis of BPH with prostate volume ≥ 80 mL and evidence of bladder outlet obstruction (BOO). All patients had transrectal ultrasound (TRUS) evidence of BPH [4]. BOO was defined based on TRUS findings of significant median lobe protrusion and/or elevated postvoid residual (PVR) urine volume. We excluded patients with prostate cancer, previous urethral or prostatic surgery, neurogenic bladder, and/or urethral stricture. In cases of suspected malignancy based on digital rectal examination (DRE), TRUS, or elevated serum prostate-specific antigen (PSA), TRUS-guided biopsies were performed, and those with confirmed cancer were excluded.

All patients underwent routine preoperative evaluation including medical history, DRE, international prostate symptom score (IPSS) questionnaire, laboratory investigations (complete blood count, coagulation profile, and renal function tests), and assessment of surgical fitness. Uroflowmetry with peak flow rate (Qmax), PVR urine, and TRUS were performed for all patients before proceeding to any surgical intervention. Prostate volume was measured using TRUS and calculated using a conventional formula (length*width*height*π/6).

### Study design, randomization and surgical procedure

This study was designed as a prospective exploratory single-center randomized trial. No formal a-priori sample-size calculation was performed. The number of participants was determined by the number of eligible patients presenting during the study period. A post-hoc power analysis was subsequently conducted using the reduction in IPSS as the primary endpoint, confirming that the sample size provided sufficient power to detect clinically significant differences in functional outcomes between the two groups. After meeting inclusion criteria and providing written informed consent, patients were randomized to undergo either B-TURP or BipolEP using a computer-generated random sequence. Allocation concealment was maintained through the use of sequentially numbered, opaque, sealed envelopes prepared by an independent statistician.

All procedures were performed using standard techniques without ejaculation-preserving modifications by four senior urologists, each with experience of more than 50 prior cases in both techniques. Cases were randomized evenly among the four surgeons to minimize operator bias and learning-curve effects. All surgeons used the same bipolar platform (Karl Storz, Tuttlingen, Germany) with a 26 Fr continuous-flow resectoscope and identical generator settings according to institutional protocol. In the BipolEP arm, the adenoma was enucleated along the capsular plane and retrieved by mechanical morcellation using a Jena Surgical morcellator (Jena Surgical, Jena, Germany). To ensure procedural consistency, a pre-study calibration session was conducted in which the surgeons standardized their operative approach regarding capsular plane identification, hemostasis, bladder neck management, and adenoma retrieval. Quality control included intraoperative supervision and regular operative audits.

### Postoperative care

After either procedure, a three-way Foley catheter was inserted for continuous bladder irrigation. Traction was applied only if urine appeared mildly hematuric and released once urine turned clear. Continuous bladder irrigation was discontinued when urine remained light pink for > 6 h and resumed if bleeding recurred. All patients were managed under an identical perioperative fluid protocol to ensure comparability between groups.

### Outcome measures

The primary endpoint was the change in IPSS from baseline to 6 months postoperatively. Secondary endpoints included changes in Qmax and PVR. Postoperative data (IPSS, PVR, and Qmax) were collected at 6 months during routine outpatient follow-up visits. Postoperative assessments were performed by a urology resident who was not involved in the surgeries and blinded to treatment allocation. Preoperative sexual function was assessed clinically through patient interview to determine baseline ejaculatory status, and postoperative retrograde ejaculation was documented during follow-up visits.

Intraoperative parameters included operative time, irrigation fluid volume, and the need for catheter traction. Postoperative measures included hospital stay, catheterization time, residual prostatic tissue, need for resuming bladder irrigation, and hemoglobin drop. Residual prostatic tissue was estimated indirectly by subtracting the resected or enucleated tissue weight from the preoperative TRUS prostate volume. Hemoglobin levels were measured preoperatively and on the first postoperative day. Postoperative complications occurring within the 6-month follow-up period were classified according to the modified Clavien–Dindo grading system.

### Statistical analysis

All analyses were conducted using Jamovi software (version 2.6.2.0). Normality of data was assessed using the Shapiro–Wilk test. Normally distributed variables are expressed as mean ± standard deviation and compared using the independent samples *t*-test. Non-normally distributed variables are presented as median and interquartile range and compared using the Mann–Whitney U test. Categorical variables are expressed as frequencies with percentages and analyzed using the Chi-squared test. A post-hoc power analysis was conducted for the primary endpoint (IPSS reduction). With an observed mean difference of 2 points (SD ~ 9), the achieved power was > 80% at α = 0.05. Comparisons of preoperative with postoperative data among each group were done using Wilcoxon rank test. To account for baseline dependence, analysis of covariance (ANCOVA) models were applied for postoperative IPSS and Qmax, adjusting for their respective baseline values. A p-value of < 0.05 was considered significant.

## Results

A total of 70 patients were enrolled, all of whom completed the 6-month follow-up and were included in the final analysis; no patients were lost to follow-up. Thirty-seven patients underwent B-TURP (group 1) and 33 underwent BipolEP (group 2). The mean age was 64.1 ± 6.56 and 67 ± 5.96 years for groups 1 and 2, respectively, with no significant differences in demographic or baseline characteristics between the two groups (Table 1).

**Table 1:** Demographic and baseline characteristics of the studied groups

	B-TURP (*n*=37)	BipolEP (*n*=33)	*P*-value
Age^*^	64.1 (6.56)	67 (5.96)	0.056^1^
Hypertension^**^	6 (16.2%)	10 (30.3%)	0.161^2^
Diabetes Mellitus^**^	5 (13.5%)	5 (15.2%)	0.845^2^
Cardiac^**^	2 (5.4%)	2 (6.1%)	0.906^2^
On antiplatelet therapy^**^	2 (5.4%)	3 (9.1%)	0.550^2^
Prostate volume^***^	100 (20)	100 (20)	0.910^3^
Median lobe^**^	25 (67.6%)	26 (78.8%)	0.292^2^

*B-TURP* bipolar transurethral resection of prostate, *BipolEP* bipolar enucleation of prostate

^*^Mean (standard deviation)

^**^ Number (percentage)

^***^Median (interquartile range)

^1^ Independent Samples T-Test, ^2^ Chi-squared test, ^3^ Mann-Whitney U test

Operative time, irrigation volume, need for postoperative catheter traction, hospital stay, catheterization time, residual prostatic tissue, and need to resume bladder irrigation were all significantly higher in group 1 (Table 2).

**Table 2:** Perioperative parameters of the studied groups

	B-TURP (*n*=37)	BipolEP (*n*=33)	*P*-value
Operative time (minutes)^*^	80.8 (12.8)	65 (13.1)	**<0.001**^1^
Amount of fluid needed for bladder irrigation during procedure (liters)^**^	34 (12)	22 (15)	**0.001**^2^
Need for catheter traction after the procedure^***^	20 (54.1%)	7 (21.2%)	**0.005**^3^
Hospital Stay (Days)^*^^*^	2 (0)	2 (0)	**<0.001**^2^
Catheterization time (Days)^*^^*^	4 (2)	2 (1)	**<0.001**^2^
Residual prostatic tissue^*^^*^	15 (20)	0 (10)	**0.004**^2^
Need for resuming bladder irrigation^**^^*^	11 (29.7%)	1 (3%)	**0.003**^3^

*B-TURP* bipolar transurethral resection of prostate, *BipolEP* bipolar enucleation of prostate

^*^ Mean (standard deviation)

^**^ Median (interquartile range)

^***^ Number (percentage)

^1^ Independent Samples T-test, ^2^ Mann-Whitney U test, ^3^Chi-squared test

The incidence of retrograde ejaculation was significantly higher in the BipolEP group. Stress urinary incontinence (SUI) was more frequent in the BipolEP group during the early postoperative period but not at 6 months, when the trend reversed, though both differences were non-significant. The incidences of hyponatremia, secondary hemorrhage, bladder neck stenosis, collection, bladder perforation, and capsular perforation did not differ significantly between the two groups. One B-TURP patient developed secondary hemorrhage one week after surgery, presenting with hematuria and clot retention. Following confirmation of hemodynamic stability, the condition was managed successfully with bladder wash, 12 h of continuous irrigation, and 24 h of observation before discharge. Another B-TURP patient developed bladder neck contracture, managed successfully with endoscopic bladder neck incision. Capsular perforation occurred in one B-TURP patient, who also developed an extraperitoneal collection that resolved with catheter drainage and inpatient observation. Two BipolEP patients developed collections secondary to bladder perforation: one extraperitoneal, managed conservatively, and the other intraperitoneal, requiring pigtail drainage removed after 36 h with complete recovery (Table 3).

**Table 3:** Complications of B-TURP and BipolEP based on the modified Clavien-Dindo classification system (6-month follow-up)

	B-TURP (*n*=37)	BipolEP (*n*=33)	*P*-value
Grade I			
Retrograde ejaculation^*^	18 (48.6%)	27 (81.8%)	**0.004**^1^
Stress Urinary incontinence at 1 month^*^	7 (18.9%)	8 (24.2%)	0.588^1^
Hyponatremia^*^	1 (2.7%)	0 (0%)	0.341^1^
Grade II			
Blood transfusion requirement^*^	7 (18.9%)	1 (3%)	**0.037**^1^
Secondary hemorrhage^*^	1 (2.7%)	0 (0%)	0.341^1^
Grade III			
Persistent stress urinary incontinence at 6 months^*^	4 (10.8%)	3 (9.1%)	0.811^1^
Bladder neck stenosis^*^	1 (2.7%)	0 (0%)	0.341^1^
Collection^*^	1 (2.7%)	2 (6.1%)	0.489^1^
Bladder perforation^*^	0 (0%)	2 (6.1%)	0.129^1^
Capsular perforation^*^	1 (2.7%)	0 (0%)	0.341^1^
Grade IV			
Grade V			

*B-TURP* bipolar transurethral resection of prostate, *BipolEP* bipolar enucleation of prostate

* Number (percentage)

^1^Chi-squared test

When comparing pre- and postoperative parameters, both groups showed significant improvements in Qmax and PVR, reflected by corresponding reductions in IPSS. However, the magnitude of IPSS reduction and Qmax improvement was significantly greater in the BipolEP group than in the B-TURP group. ANCOVA analysis adjusting for baseline IPSS and Qmax confirmed that the between-group differences remained significant for both postoperative IPSS (*p* = 0.006) and postoperative Qmax (*p* = 0.005). Hemoglobin levels decreased significantly in both groups, but the decline was greater, and postoperative levels were lower in the B-TURP group (Table 4).

**Table 4:** Comparison of preoperative with postoperative parameters of the studied groups (postoperative data collected at 6 months)

Variable		B-TURP (*n*=37)	BipolEP (*n*=33)	*P*-value
IPSS	Preoperative^*^	29 (6)	28 (8)	0.603^1^
	Postoperative^*^	9 (7)	6 (7)	**0.007**^1^
	Preoperative to postoperative^*^	19 (9)	21 (9)	**0.040**^1^
	*P*-value of preoperative to postoperative	**<0.001**^2^	**<0.001**^2^	
PVR	Preoperative^*^	106 (150)	70 (150)	0.627^1^
	Postoperative^*^	0 (40)	0 (0)	**0.010**^1^
	Preoperative to postoperative^*^	87 (150)	150 (150)	0.222^1^
	*P*-value of preoperative to postoperative	**<0.001**^2^	**<0.001**^2^	
Qmax	Preoperative^*^	5 (3.22)	5.20 (5.40)	0.229^1^
	Postoperative^*^	14.60 (6.80)	19.50 (10.6)	**0.004**^1^
	Preoperative to postoperative^*^	−11.9 (7.10)	−16.1 (7.10)	**0.003**^1^
	*P*-value of preoperative to postoperative	**<0.001**^2^	**<0.001**^2^	
Hb level	Preoperative^*^	13.30 (1.70)	12.80 (2.40)	0.187^1^
	Postoperative^*^	10.50 (1.30)	11.20 (2.60)	**0.009**^1^
	Preoperative to postoperative^*^	2.80 (2.20)	1.50 (1)	**0.002**^1^
	*P*-value of preoperative to postoperative	**<0.001**^2^	**<0.001**^2^	

*B-TURP* bipolar transurethral resection of prostate, *BipolEP* bipolar enucleation of prostate, *IPSS* international prostate symptom score, *PVR* post-voiding residual urine, *Qmax* peak flow rate, *Hb* Hemoglobin

^*^ Median (interquartile range)

^1^ Mann-Whitney U test

^2^ Wilcoxon rank test

## Discussion

Although TURP has long been regarded as the gold standard for surgical management of BPH [5, 6], it is not without limitations [7, 8]. These limitations prompted numerous procedural modifications. Historically, monopolar TURP was associated with morbidity rates of 15–18% [9], leading to the introduction of B-TURP, which allows the use of saline irrigation and thereby largely eliminates the risk of TURS [9–11]. Nevertheless, even with B-TURP, limitations remained in managing large prostates [8]. To address this, Liu et al. introduced BipolEP, in which the adenoma is enucleated using a bipolar plasma kinetic resectoscope in a manner similar to open transvesical prostatectomy [12]. However, BipolEP did not initially achieve the same level of adoption as B-TURP, with some authors arguing that its advantages were minimal [7].

The current study was conducted to determine whether BipolEP provides measurable advantages over B-TURP in patients with prostates ≥ 80 mL. Our findings indicate that BipolEP yields superior functional outcomes and more favorable perioperative parameters. Improvements in IPSS and Qmax were greater, and perioperative measures such as operative time, catheterization duration, and tissue removal favored BipolEP. Retrograde ejaculation was more common in the BipolEP group, while the rates of other complications did not differ significantly.

Intraoperatively, the B-TURP group had longer operative time, greater irrigation fluid use, and a higher need for catheter traction, reflecting the greater risk of bleeding. These findings align with previous reports by Magistro et al. [8] and Wei et al. [7]. The greater bleeding tendency in B-TURP is further evidenced by the higher transfusion rate, greater hemoglobin drop, and lower postoperative hemoglobin levels. This can be explained by the surgical technique: BipolEP dissects along the relatively bloodless plane of the surgical capsule after devascularizing the adenoma [12, 13], whereas B-TURP involves cutting through vascular tissue until reaching the capsule [14]. Similar observations were reported by Zhang et al. [14] and Liu et al. [12].

Postoperatively, hospital stay, catheterization duration, and the need for resuming bladder irrigation were all higher in the B-TURP group, likely due to the greater intraoperative bleeding risk. Comparable findings were reported by Magistro et al. [8] and Wei et al. [7]. Residual prostatic tissue was significantly higher after B-TURP, consistent with the more complete adenoma removal achieved in BipolEP, which follows the anatomic plane of the surgical capsule. Magistro et al. [8] likewise demonstrated greater tissue removal with BipolEP and a longer catheterization time after B-TURP.

Regarding complications, there were no significant differences between groups in the incidences of SUI, hyponatremia, secondary hemorrhage, bladder neck stenosis, collection, bladder perforation, or capsular perforation. However, retrograde ejaculation was significantly more frequent after BipolEP, and blood transfusion was more common after B-TURP. Although SUI could theoretically occur more often after BipolEP due to the proximity of the sphincter during enucleation, our results showed no significant difference in SUI rates at one or six months postoperatively. Bipolar resection with saline irrigation effectively prevents dilutional hyponatremia; the single mild case of hyponatremia observed likely resulted from excessive intraoperative absorption (hyperhydration) rather than TURS. Wei et al. [7] similarly reported no differences between TURP and BipolEP in hyponatremia or bladder neck stenosis, though they observed a higher risk of postoperative incontinence after TURP. The higher incidence of retrograde ejaculation in the BipolEP group may be explained by greater tissue removal at the bladder neck during enucleation.

When comparing preoperative with postoperative outcomes, both groups demonstrated significant improvement in IPSS, Qmax, and PVR. However, between-group analysis revealed that BipolEP achieved a higher postoperative Qmax and lower PVR than B-TURP. While this trend may partly explain the lower postoperative IPSS observed in the BipolEP group, we acknowledge that symptom severity and obstruction parameters (Qmax, PVR) do not always correlate directly. Therefore, this interpretation should be considered an observation rather than a causal relationship. Notably, Magistro et al. [8] reported no significant differences between TURP, BipolEP, and HoLEP in postoperative Qmax, PVR, and IPSS, possibly due to their inclusion of patients with smaller prostates compared with our cohort of large-gland cases.

## Limitations and recommendations

Although the study was adequately powered to detect differences in functional outcomes, it was underpowered to assess uncommon complications such as capsular or bladder perforation. Larger multicenter studies are therefore needed to validate these safety findings. We acknowledge that no pre-trial sample-size calculation or minimal clinically important difference (MCID) for IPSS was performed, as the study was exploratory in nature. Although cases were evenly randomized among surgeons, clustering analysis by operator was not performed because of the limited sample size. In addition, residual prostatic tissue was estimated indirectly by comparing resected tissue weight with preoperative TRUS volume, as postoperative imaging was not routinely performed. Another limitation is that sexual function was not evaluated using a validated questionnaire as the primary focus of the study was on voiding and perioperative outcomes.

## Conclusions

Both B-TURP and BipolEP produced significant improvement in IPSS, Qmax, and PVR at six months postoperatively. However, BipolEP achieved greater improvement in IPSS and Qmax, with shorter operative time, hospital stay, and catheterization duration, and markedly less intraoperative bleeding. Retrograde ejaculation was more frequent after BipolEP, representing the main trade-off. Overall, BipolEP appears to be a safe and effective option and may be preferred for the surgical management of BPH in patients with large prostate volumes.

## Supplementary Information

Supplementary Material 1.

## Data Availability

The datasets generated and/or analyzed during the current study are available from the corresponding author on reasonable request.
