# Supplementary material for: Bipolar resection versus enucleation of the prostate in management of benign prostatic hyperplasia patients with large-sized prostates: a prospective randomized controlled clinical trial
Source: BMC Urol. 2026 Jan 24;26:52. doi: 10.1186/s12894-025-02004-1 (PMC12930823; doi:10.1186/s12894-025-02004-1)
Supplement: Supplementary file 1 — Supplementary Material 1. [file 12894_2025_2004_MOESM1_ESM.docx]

**Title:**

**Bipolar resection versus enucleation of the prostate in management of benign prostatic hyperplasia patients with large-sized prostates: A prospective randomized controlled clinical trial**

**Running Title:**

B-TURP vs BipolEP in large prostates

**Authors and Affiliations:**

**1-Mostafa M. Mostafa, MD, MSc, PhD**

Lecturer of Urology,

Assiut University Hospitals and college of Medicine,

Asiut, Egypt,

E-mail: [mostafaabdelaziz91@gmail.com](mailto:mostafaabdelaziz91@gmail.com)

Phone number: +2 01000740478

ORCID ID: 0000-0002-8750-6236

**2- Ahmed F. Ibrahim, MD, MSc**

Assistant Lecturer of Urology,

Assiut University Hospitals and college of Medicine,

Asiut, Egypt,

E-mail: [ahmedfarahat@aun.edu.eg](mailto:ahmedfarahat@aun.edu.eg)

Phone number: +2 01069569946

**3-Ahmed M. Moeen, MD, MSc, PhD**

Professor of Urology,

Assiut University Hospitals and college of Medicine,

Asiut, Egypt,

E-mail: [ahmedmoeen@aun.edu.eg](mailto:ahmedmoeen@aun.edu.eg)

Phone number: +2 01003960931

**4-Mohammed A. Elgammal, MD, MSc, PhD**

Professor of Urology,

Assiut University Hospitals and college of Medicine,

Asiut, Egypt,

E-mail: [mohammedelgammal@aun.edu.eg](mailto:mohammedelgammal@aun.edu.eg)

Phone number: +2 01005212163

**5-Ahmed S. El-Azab, MD, MSc, PhD**

Professor of Urology,

Assiut University Hospitals and college of Medicine,

Asiut, Egypt,

E-mail: [elazab@aun.edu.eg](mailto:elazab@aun.edu.eg)

Phone number: +2 01001011071

**6-Mohammad A. Faragallah, MD, MSc, PhD**

Lecturer of Urology,

Assiut University Hospitals and college of Medicine,

Asiut, Egypt,

E-mail: [abas@aun.edu.eg](mailto:abas@aun.edu.eg)

Phone number: +2 01002438659

**Corresponding Author:**

**Mostafa M. Mostafa, MD**

Lecturer of Urology,

Assiut University Hospitals and college of Medicine,

Asiut, Egypt,

E-mail: [mostafaabdelaziz91@gmail.com](mailto:mostafaabdelaziz91@gmail.com)

Phone number: +2 01000740478

ORCID ID: 0000-0002-8750-6236

**Declarations**

**Ethics approval and consent to participate:**

Approval was obtained from the Assiut University College of Medicine Institutional Review Board (IRB number: 17200747). The authors also certify that the study was performed in accordance with the ethical standards as laid down in the 1964 Declaration of Helsinki and its later amendments.

The nature of the study, its aim, the procedures performed, and any risk from participation in the study were explained in detail to the patients before enrolling in the study, and patients who voluntarily agreed to participate in the study signed an informed consent prior to participating in the study.

**Availability of Data and Materials:**

The datasets generated and/or analyzed during the current study are available from the corresponding author on reasonable request.

**Consent for publication:**

Written informed consent for publication was obtained from patients involved in this study.

**Clinical trial registration**

The study is registered in clinical trials (ClinicalTrials gov ID: NCT05330156 ; Registered on April 15, 2022).

**Word count of text:**

2193

**Word count of abstract:**

254

**Conflicts of Interest:**

The authors have no conflicts of interest to disclose.

**Acknowledgments:**

None declared

**Funding:**

This research received no specific grant from any funding agency in the public, commercial, or not-for-profit sectors.

**Authors’ contributions:**

Research conception and design: MM Mostafa, AF Ibrahim, AM Moeen, MA Faragallah. Data acquisition: AF Ibrahim, AS El-Azab, MA Faragallah. Statistical analysis: MM Mostafa, MA Elgammal. Data analysis and interpretation: MM Mostafa, AM Moeen, MA Elgammal. Drafting of the manuscript: MM Mostafa, AF Ibrahim, MA Faragallah. Critical revision of the manuscript: AM Moeen, MA Elgammal, AS El-Azab. Obtaining funding: N/A. Administrative, technical, or material support: MM Mostafa, AM Moeen, MA Faragallah. Supervision: MA Elgammal AS El-Azab. Approval of the final manuscript: all authors.
